# Retrospect of the Medical Sciences

**Published:** 1843-02-18

**Authors:** 


*Successful Operation for Strangulated Hernia in an Infant seven weeks old.* By Mr. CÆSAR HAWKINS.

Charles Tunstall, æt. seven weeks, was admitted into St. George's Hospital, Feb. 21, 1840, with oblique inguinal hernia, which had been strangulated two days, during which time there had been no evacuation. The child had constantly vomited when put to the breast, and the countenance was pallid and sunk, and expressive of severe suffering The tumor was of considerable size for so small a child, and tense, though not externally inflamed. Attempts had been made to reduce the tumor before the child's admission, with the assistance of a warm bath: castor oil had been administered, and one leech applied. The mother was not aware that the hernia existed at the time of birth, but she had observed the child cry whenever the part had been washed or touched.

It was obvious that no time could be lost, and I immediately performed the operation, and returned the intestine, which was little inflamed; the sac was very thick, and contained a good deal of fluid.

An evacuation took place an hour after the operation, and the next day, February 22d, there had been no return of sickness, and though the child refused the breast it had taken a little arrow-root; the countenance was also improved; the tongue moist, and the pulse had tolerable force, though it was rather slow and irregular; the abdomen was soft, but pressure made the child cry. Relief was given by a chamomile poultice.

23d. This tenderness had gone, and the child again sucked readily, and the wound looked healthy.

On the 25th I was rather anxious on account of the urine, which had made the wound tumid and inflamed; the sloughing was confined, however, to the cellular membrane and the outer part of the sac, and the wound afterwards healed well.

I saw the child a few months ago, at which time a considerable sized hernia existed under the cicatrix.

I have reason to believe that an opinion is commonly entertained by medical men that hernia is attended with little danger in infants; but although, doubtless, not so liable to strangulation as in older persons, the opinion leads to grave errors in practice, if it is neglected. On this account I conceive it is the right practice to endeavour to obliterate the canal at the earliest period by the gentle pressure which can be applied in the youngest infant by the common belt and pad, and understrap, or by a small spring truss:—

1st. Because strangulation may take place, as in the case above related, so as to require an operation, which might probably have been prevented, if pressure had been used to keep the bowel up. In two instances I have seen strangulation at the early period of three weeks, and in one of these cases reduction was effected with much difficulty by means of ether dropped constantly on the tumor for about an hour and a half: there was much sickness in this case, and a good deal of tenderness of the tumor, and it was clear that I could not have delayed the operation very long. In another case an operation was performed by another surgeon of the hospital, in a child about two years old, but it was followed by fatal inflammation.

2d. By using pressure at an early period, the surgeon takes advantage of the natural disposition to obliteration of the canal, which exists at the time of the descent of the testis, so that a cure may be effected in four or six weeks, which will require as many months at a later period; and,

3d. The difficulty of the cure is very much increased by the enlargement of the canal from frequent protrusion of the bowel. A few months ago, I was consulted by a lady whose child was ten months old, with a hernia of three months' duration, which had increased considerably in size, and was very difficult to keep up by a truss: she had been advised by a provincial surgeon of much repute to do nothing for the hernia, but as her usual attendant saw it increasing, I was written to on the subject, and I think much trouble might have been saved if a truss had been used as soon as the hernia had been discovered.

Some countenance is supposed to be given to the common opinion, by the authority of Sir Astley Cooper; who does not, in fact, however, recommend this delay, except in the few cases in which I should agree with him in postponing the treatment, viz.: in cases in which the testis has not descended into the scrotum, but lies in so inconvenient a position in the groin, as to prevent the application of a truss. Even in these cases, however, attention should be paid to the further progress of the testis, so that the pressure may be delayed, not to the time of puberty, but only to the earliest period at which this gland may happen to have descended low enough to allow the truss to be used.—*London Medical Gazette*, December 30, 1842.

---

FRACTURE OF THE CLAVICLE WITHOUT DISPLACEMENT.

In some cases of fracture of the clavicle occurring about the middle of the bone in young subjects, displacement of the fragments does not immediately take place, thus giving rise to a risk of an error in diagnosis, by which the ultimate probability of a cure is diminished. A lad, seventeen years of age, was recently admitted into the Hôtel-Dieu, under the care of M. Blandin, having a few days previously, fallen upon one of his comrades while playing with him, when he instantly experienced pain, and a cracking sensation about the middle of the left clavicle, where there soon formed a tumor, which, increasing, induced him to enter the hospital. On examination, the swelling was found to occupy the middle of the clavicle; it was about as large as half a hen's egg, ovoid in shape, well circumscribed, colorless, and hard, but sensible to pressure. There was not any deformity of the shoulder, nor any abnormal modification of the axis of the bone, to indicate the existence of a fracture, and although the different movements of the arm caused pain in the shoulder, yet they could be made without much difficulty.

The symptoms in this case would lead to the belief that it was a case of simple periostitis, caused by external violence; but M. Blandin at once decided that there existed a fracture of the bone, having seen a similar case previously at the hospital Beaujon, where the tumour was treated as traumatic periostitis, the patient merely carrying his arm in a sling, until, by a sudden movement of the limb, displacement of the fragments was produced, and clearly demonstrated the existence of a fracture. A second case occurring soon afterwards, M. Blandin profited by the experience gained from the preceding, and by moving the fragments of the broken clavicle on each other, obtained motion and crepitus. Still these indications were not so clear that M. Marjolin could diagnosticate a fracture; he was of opinion that the case was one of exostosis, probably syphilitic, and the crepitus he believed depended on an erosion of the osseous surface. In consequence, the patient was left to himself, until a movement of the arm gave proof of the fracture by the displacement of the broken portions of the bone.

Two other cases occurring in young subjects have been admitted since into the Hôtel-Dieu under the care of M. Blandin, one of whom was purposely left without surgical assistance, while Dessault's bandage was applied to the other. The former soon showed evidence of consecutive displacement; the latter was cured without any deformity following.

The surgeon may diagnose a fracture, without displacement, of the middle portion of the clavicle, when a circumscribed tumor forms in that part in young subjects, consecutive on a fall on the shoulder, and motion of the fragments with crepitus can be detected, there not being any syphilitic taint in the constitution.

M. Blandin considers the elasticity of the periosteum, and the cartilaginous layer covering the bone in young subjects, as the cause of the broken portions of the bone not separating from each other until after a fresh action on the part overcomes the resistance offered by the fibrous envelope. The five patients who came under his notice with this accident were all under twenty years of age.—*Prov. Med. Journ., from Journ. de Med. et. Chir. Prat.*, July, 1842.

---

### THE DISEASES OF NIGHTMEN.

A communication from MM. Bricheteau, Chevalier, and Furnari, has been published on the diseases to which nightmen are liable, from which it appears that this mode of gaining a subsistence, although dirty and disgusting, is less unhealthy than many others that may easily be named; the workmen continue to practice it to a great age (from 20 to 65 or 70;) that it is probably curative or preservative from certain diseases of the skin; and, lastly, nightmen are less exposed than many other workmen to attacks of epidemic diseases. The statements thus made are drawn from direct observation, from the replies of the masters, and other sources. The men themselvss are generally healthy, strong, and vigorous, and their children, equally healthy, do not manifest any repugnance to their parents' employment.

The two chief complaints to which these men are subject, arising from the nature of their occupation, are—a peculiar ophthalmia resulting from the direct influence of the deleterious gases disengaged in cesspools on the eye, the effects of which are never permanent, and the asphyxia produced by the inhalation of the same gases. To this latter affection nightmen are less subject than masons who are engaged a few days after the emptying of the cesspool to repair the brickwork; no cases of the kind would occur if proper care were taken to ventilate the cesspool, and to clear out the gases previous to the men going down into it. That it can easily be avoided is proved by the fact, that a master nightman who employs annually from eight to twelve nightmen and masons, has not had a case of asphyxia among them for twenty years, and another who has from eight to ten men daily at work, has had only one case, whieh was cured in ten hours; and a third master, employing fifteen men, including carters, had also never had a case. The most dangerous periods are at the commencement and termination of the work, and some few days after the cesspool has been emptied; the exhalations are more active, and accidents more frequent in the summer and autumn than in winter or spring.—*Prov. Med. Journ. from Annales d' Hygiène Publique*, July, 1842.

---

### MORBID ANATOMY OF THE NERVOUS SYSTEM.

On examining "Schmidt's Journal," No. 487, we find, in a paper on the Morbid Anatomy of the Nervous System and the Organs of Sense, that much attention has recently been devoted by M. Gluge to determining the morbid appearances in the above portions of the frame by the aid of the microscope. The appearances in the brain after death from acute hydrocephalus do not afford, he says, any indications of previous inflammation. The serosity exuded into the ventricles contains but little albumen, and the softening of the brain in the parts contiguous appears to be solely an effect of maceration. The red points scattered throughout the cerebral substance in cases of congestion, M. Gluge has ascertained to be due to an extraordinary number of capillary vessels compactly united with each other, and which he regards as vessels of new formation. Apoplexy, he says, is always attended with sanguineous effusion, the ruptured vessels being in some cases the capillaries, in others the larger arteries. The apoplectic nucleus, examined immediately, or a few days after death, is found to consist of a coagulum of red globules; of fibrin, seldom much consolidated; and of the remains of capillary vessels, commonly filled with coagulated blood. The white filmy shreds inclosed in the clot are not false membranes, the products of inflammation, but broken down cerebral substance.—*Lond. Lancet.* Dec. 31.

---

### IRIDOPLASMA.

*Iridoplasma* is a name given by M. Gluge to a degenerescence of the eye, the structures of which assume colours that are very different from those which they have in their natural state. In one instance that he has reported the eye consisted of a succession of structures of four different colours. The first was composed of a substance which was similar, both in consistence and colour, to the brain of a newly born infant, inclosing many whitish irregular bodies. The second and third layers were pale, or of a yellowish-white tinge, with here and there similar irregular bodies scattered through their substance. The fourth structure was the crystalline lens, unchanged from the normal condition as regards its composition and transparency, but coloured green. Small fatty bodies were dispersed throughout each of these structures, all of which were enclosed in the sclerotic coat without being adherent to it. There was no trace of either a retina or a vitreous humour, and some blackish striæ alone were supposed to represent the choroid coat. The optic nerve had its natural form, but instead of true nervous matter, it consisted of a yellow substance, which, under the microscope, presented an appearance similar to that of the second and third layers in the eyeball above described.—*Ibid.*

